# Adoptive T Cell Therapy Strategies for Viral Infections in Patients Receiving Haematopoietic Stem Cell Transplantation

**DOI:** 10.3390/cells8010047

**Published:** 2019-01-14

**Authors:** Giorgio Ottaviano, Robert Chiesa, Tobias Feuchtinger, Mark A. Vickers, Anne Dickinson, Andrew R. Gennery, Paul Veys, Stephen Todryk

**Affiliations:** 1 Department of Paediatrics, Milano-Bicocca University, Fondazione MBBM/San Gerardo Hospital, 20900 Monza, Italy; 2 Blood & Marrow Transplant Unit, Great Ormond Street Hospital, UCL, London WC1N 3JH, UK; robert.chiesa@gosh.nhs.uk (R.C.); paul.veys@gosh.nhs.uk (P.V.); 3 Department of Pediatric Hematology, Oncology, Hemostaseology and Stem Cell Transplantation, Dr. von Hauner University Children’s Hospital, LMU, 80337 Munich, Germany; tobias.feuchtinger@med.uni-muenchen.de; 4 Scottish National Blood Transfusion Service, Blood Transfusion Centre, Foresterhill Road, Aberdeen AB25 2ZW, UK; m.a.vickers@abdn.ac.uk; 5 Infection, Immunity & Inflammation, Institute of Medical Sciences, University of Aberdeen, Aberdeen AB25 2ZD, UK; 6 Haematological Sciences, Newcastle University, Newcastle upon Tyne NE2 4HH, UK; anne.dickinson@newcastle.ac.uk; 7 Institute of Cellular Medicine, Newcastle University, Newcastle upon Tyne NE2 4HH, UK; andrew.gennery@newcastle.ac.uk (A.R.G.); stephen.todryk@northumbria.ac.uk (S.T.); 8 Paediatric HSCT Unit, Great North Children’s Hospital, Newcastle upon Tyne NE2 4HH, UK; 9 Department of Applied Sciences, Faculty of Health & Life Sciences, Northumbria University, Newcastle upon Tyne NE1 8ST 4HH, UK

**Keywords:** haematopoietic stem cell transplantation, viral infections, adoptive cell therapy, third party donor

## Abstract

Adverse outcomes following virus-associated disease in patients receiving allogeneic haematopoietic stem cell transplantation (HSCT) have encouraged strategies to control viral reactivation in immunosuppressed patients. However, despite timely treatment with antiviral medication, some viral infections remain refractory to treatment, which hampers outcomes after HSCT, and are responsible for a high proportion of transplant-related morbidity and mortality. Adoptive transfer of donor-derived lymphocytes aims to improve cellular immunity and to prevent or treat viral diseases after HSCT. Early reports described the feasibility of transferring nonspecific lymphocytes from donors, which led to the development of cell therapy approaches based on virus-specific T cells, allowing a targeted treatment of infections, while limiting adverse events such as graft versus host disease (GvHD). Both expansion and direct selection techniques have yielded comparable results in terms of efficacy (around 70–80%), but efficacy is difficult to predict for individual cases. Generating bespoke products for each donor–recipient pair can be expensive, and there remains the major obstacle of generating products from seronegative or poorly responsive donors. More recent studies have focused on the feasibility of collecting and infusing partially matched third-party virus-specific T cells, reporting response rates of 60–70%. Future development of this approach will involve the broadening of applicability to multiple viruses, the optimization and cost-control of manufacturing, larger multicentred efficacy trials, and finally the creation of cell banks that can provide prompt access to virus-specific cellular product. The aim of this review is to summarise present knowledge on adoptive T cell manufacturing, efficacy and potential future developments.

## 1. Introduction

Haematopoietic stem cell transplantation (HSCT) represents an important curative option for a large group of malignant (mainly leukaemias and lymphomas) and nonmalignant disorders (e.g., primary immunodeficiencies and metabolic diseases). However, outcomes can be hampered by a wide spectrum of transplant-related complications including viral infections, which are a major cause of morbidity and mortality in transplanted patients [1]. Accurate incidences of viral infections in transplant settings are not reported consistently since differences in sample analysis (e.g., whole blood versus plasma) [2] or different viral load cut-offs for positivity, can lead to heterogeneous results [3]. Although pharmacological therapies are available to treat viral infections, many are ineffective due in part to drug resistance, or having to cease therapy due to drug-related toxicities. Furthermore, prolonged therapy is expensive. For these reasons, virus-specific T cells (VsTs), mainly cytotoxic T lymphocytes (CTLs), have been increasingly investigated as a treatment option for refractory viral infections in transplanted patients. 

Different strategies for VsT manufacture have been employed to improve viral specificity of T cells towards single or multiple viruses, including methods of cell selection or in-vitro stimulation, choice of cell source, and HLA (human leukocyte antigen) matching. In this review, we illustrate the relevant differences in approaches to adoptive cell therapy using lymphocytes, focusing on factors influencing the efficacy of CTLs, and overview the latest advances and possible future developments of antiviral T-cell therapy.

## 2. Refractory Viral Infections Following HSCT

Preemptive therapy for viral infections in transplanted patients aims to treat subclinical viral reactivation before clinical manifestations appear, since during the immunocompromised state of transplanted patients there is insufficient host immunity to control viral replication. The first-line approaches to viral infections comprise tapering of immunosuppression, and antiviral drug therapy. However, patients may not respond due to the lack of immune reconstitution, viral drug resistance or drug toxicity. Patients receiving serotherapy as part of conditioning (to deplete T cells), or steroids for treatment of GvHD, are at higher risk of viral reactivation. Prophylaxis against GvHD with T cell-depleting alemtuzumab can lead to a profound lymphopenia that raises the risk of viral infection. Data on the impact of HLA matching show that mismatched transplants are not usually associated with a higher susceptibility to infections [4]. Routine monitoring of viral reactivation in the post-transplant setting usually includes molecular detection of viral DNA of the three most frequent viruses responsible for refractory infections, namely human adenovirus (AdV), cytomegalovirus (CMV) and Epstein–Barr virus (EBV). Data on incidence of viral reactivation, viral disease, standard treatment and rate of response are summarised in Table 1. AdV-associated disease usually occurs within the first two months post-transplant. Although some patients remain asymptomatic and clear the virus spontaneously, others can present with rapid progression to fatal multiorgan failure. The incidence of AdV viremia following HSCT is higher in paediatric patients, with a significantly higher mortality for patients developing AdV disease [5]. Clinical manifestations include haemorrhagic enteritis or cystitis, pneumonia, hepatitis, encephalitis and multiorgan failure [6]. Cidofovir is currently the recommended first-line drug for pre-emptive therapy of AdV infection [7], although outcome is usually hampered by T-cell lymphocytopenia and renal toxicity. However, the evidence for reducing mortality is inconsistent, with similar mortality rates (~ 20%) being reported in patients receiving or not receiving pre-emptive therapy [8,9]. For these reasons, new pharmacological approaches have been explored, leading to the development of new molecules. Brincidofovir has been recently adopted for refractory AdV infections with good rates of response, although data remain scarce, especially in children [10]. Moreover, this drug is also associated with some organ toxicity, mainly related to the gastrointestinal tract. In a recent retrospective study, brincidofovir appeared to reduce adenoviral load more rapidly than cidofovir, and did so in the absence of lymphocyte recovery [11]. Prospective studies are underway to assess the effect of brincidofovir on adenoviral-related mortality following HSCT (AdAPT).

Cytomegalovirus reactivation following HSCT can occur in up to 40% of transplanted patients [12], although pre-emptive pharmacological approaches reduce incidence of CMV disease. Gastrointestinal and pulmonary involvement are the main clinical presentations, but evolution to disseminated disease can occur [13]. Several drugs can be used to tackle CMV reactivation. The best established is ganciclovir, although associated myelotoxicity precludes its use as first-line therapy in the early phases of transplants. Foscarnet is generally the next alternative to ganciclovir for CMV infections at this stage, although it is associated with a significant risk of renal toxicity. There is some early data on the use of oral valganciclovir in the bone marrow transplant setting, but myelotoxicity may still be a problem [14,15]. Akin to bacterial resistance to antibiotic therapy, recent next-generation sequencing studies are now highlighting the role of CMV genome mutations resulting in drug-resistant virus strains [13,16].

Epstein–Barr virus (EBV) reactivation after HSCT can lead to clonal proliferation of CD20^+^ B cells, potentially causing lymphoproliferative disease. The increased use of anti-CD20 monoclonal antibody (Rituximab) has significantly reduced the incidence and mortality of EBV-driven post-transplant lymphoproliferative disease (PTLD), especially in children [17]. However, although anti-CD20 therapy can lead to excellent response rates as a pre-emptive strategy, efficacy as treatment of PTLD is around 60% [18]. Indeed, occasional patients are refractory to Rituximab, and require consideration for chemotherapy (e.g., CHOP) or adoptive CTLs. 

Human herpes virus 6 (HHV6) can also cause occasional severe organ failure (lung, liver, CNS) and, rarely, fatal outcome in post-transplant patients. Viral reactivation needs to be distinguished from chromosomal integration. Risk factors for viral reactivation include delayed T-cell reconstitution, co-reactivation with other viruses, and use of cord blood as a source of stem cells [19], although the exact prevalence of HHV6 reactivation is not well documented since it is not part of the routine viral monitoring in transplanted patients, and may be found in the absence of any associated clinical features. Therapy with ganciclovir, foscarnet and cidofovir have been variously used to tackle HHV6 reactivation, but there is no consensus on therapeutic, prophylactic or preventive strategies.

Hemorrhagic cystitis due to BK virus infection is a painful and difficult condition to treat, and can hamper the outcome of HSCT. Although some authors reported that cidofovir either systemic or intravesical can improve or reduce BK viremia or viruria, there are insufficient data to recommend its use as standard treatment.

Progressive multifocal leukoencephalopathy (PML) is a severe potentially fatal complication caused by JC virus reactivation, which can occur usually in late post-transplant phases (11–60 months). The diagnosis represents a significant challenge for clinicians as there are no specific treatments to prevent or reduce progression. Cidofovir, IL-2, cytarabine and IVIG have been variably used, but there is little evidence to suggest that such therapies prevent fatal outcome, which eventually occurs in a considerable proportion of patients.

Antiviral responses are mainly related to the patients’ adaptive immune recovery, which is unlikely to have recovered in the early days after transplantation [20]. Post-transplant immune reconstitution can be influenced by several variables, mainly related to transplant characteristics. Use of in vitro or in vivo T cell depletion is universally accepted as a main strategy to avoid graft rejection and reduce graft versus host disease for transplants from unrelated or mismatched family donors. However, this results in a consistent delay in recovery from profound lymphopenia. Anti-thymocyte globulin (ATG), derived from either rabbit or horse sera, specifically targets T cells, while anti-CD52 monoclonal antibody (Alemtuzumab) depletes T and B lymphocytes as well as NK cells. The risk of viral infections is generally higher using Alemtuzumab due to delayed lymphocyte recovery, when compared to ATG [21], although timing and dosing of each antibody will play a major role.

HLA matching can also impact on the rapidity of lymphocyte recovery post-transplant, and a slower T cell recovery usually occurs after haploidentical transplantation [22]. The stem cell source is also responsible for the speed of lymphocyte recovery. Peripheral blood or bone marrow stem cells generally give a faster increase in the number of T and B lymphocytes, as compared to cord blood. However, omission of in vivo T cell depletion with ATG in cord blood unit recipients leads to a unique and rapid expansion of CD4^+^ lymphocytes [23].

Viral strains can show mutation in viral DNA that can confer resistance to pharmacological treatment, although most refractory viral infections do not show a specific pattern of DNA mutations, and no consensus on patient selection for genotypic drug resistance testing is currently available [24].

## 3. From DLI to Virus-Specific T Cells and Beyond

Therapy with ex vivo-selected lymphocytes to treat viral infections after HSCT was originally based on infusion of nonspecific donor-derived lymphocytes (donor lymphocyte infusions: DLIs). The pioneering studies of this approach proved that restoring antiviral immunity in immunocompromised hosts was feasible and could cure refractory viral infections [25,26]. Good response rates were first observed in patients treated for EBV-driven post-transplant lymphoproliferative disease (PTLD) [25]. However, the broad T cell receptor repertoire of infused T cells, not specifically committed to antiviral activity, but also targeting host MHC, led to development or exacerbation of GVHD. This seemed to limit the use of DLI in transplanted patients, but refinement of the cellular product, through depletion of allo-reactive T cells [27,28], was implemented in order to reduce the risk of GVHD, yet preserving antiviral activity. 

Further development of cellular therapies for viral infections focused on the specificities of T cells for different viruses, aiming to achieve higher response rates [29,30]. The first and most widely used protocols to develop virus-specific T cells (VSTs) were based on ex vivo generation and in vitro expansion of T cells, leading to a final product comprising polyclonal T cells (recognizing different immunogenic viral antigens), with the manufacture of substantial numbers of cells from a relatively small volume of blood. More importantly, one of the main advantages of ex vivo differentiation of VSTs is that it could overcome the potential obstacle represented by paucity of specific immunity for the virus in the donor immune system. However, this approach can require a long production time (from a minimum of 13 days and up to 3 months), which can limit the use of ex vivo-expanded VSTs in critically ill patients [31,32].

A more rapid way to obtain VSTs from the donor is direct selection of specific T cells, using either (1) viral peptide multimers conjugated to magnetic beads, to select highly pure cytotoxic T cells, or (2) stimulation with viral peptides followed by IFN-gamma capture assay with magnetic beads, which allows selection of both CD4^+^ and CD8^+^ virus-specific T cells. CD4^+^ T cell help is considered essential to induce a sustained immune response, whereas CD8^+^ T cells are responsible for the rapid antiviral effects. Therefore, a cellular product containing both CD4^+^ and CD8^+^ T cells is thought most likely to rapidly impart sustained protective immunity. These two approaches enable rapid production of cellular products specifically directed against viruses [33,34], although these techniques are mainly limited to donors that have already developed a specific immunological memory for the virus. Moreover, a large volume of donor blood (100–500 mL) is usually needed to obtain clinically useful cell doses. Furthermore, in vitro activation or expansion can exhaust the resulting T cells. 

There have been no large controlled prospective randomized trials using good manufacturing practice (GMP) processes investigating the efficacy of VSTs obtained by either exvivo expansion or direct selection, though there have been many small-scale studies (Table 2). However, there are now plans for a multinational, placebo-controlled, Phase-III clinical trial (TRACE (transfer of multivirus-specific T cells), involving some of the authors, led by T.F.) which will aim to generate evidence so that adoptive transfer of virus-specific T cells can be included into evidence-based treatment guidelines to become a standard treatment for refractory viral infections post-HSCT. 

Manufacturing virus-specific T cells is based upon knowledge of viral antigen immunogenicity. While some viral antigens capable of inducing an immunological response are well characterized (e.g., pp65 for CMV, EBNA2–6 for EBV), this is less understood for other viruses, such as AdV, HHV6 and BK virus. Dedicated BKV-specific T cell products manufactured using peptides and rapid antigen capture based on multimers have been described [40,41]. There is only one report on the use of ex vivo-specific CTLs for JC PML [42], which showed the proof of principle of this approach. Currently, a National Institutes of Health (NIH) Phase I study (NCT02694783) is recruiting patients for use of JC virus-specific CTLs in transplanted patients with PML.

Post-transplant management of viral infections can be particularly challenging in the presence of multiple refractory viral infections, and in order to obtain control of multiple infections with adoptive T cells, patients can be treated with multivirus-specific T cell therapy [32] (Table 3). The rationale is based on recognition by CTLs of immunodominant viral proteins derived from CMV, EBV and AdV simultaneously. This approach could incorporate targeting other viruses, such as HHV6 and BK virus [43]. The different immunogenic potentials of viral antigens could lead to antigen competition during in vitro manufacturing of CTLs, resulting in loss of specificity towards less immunogenic viral epitopes (such as AdV) in the final product. However, it has been shown that low frequencies of infused donor-derived CTLs can sufficiently expand in vivo with clinical benefit [44].

## 4. Efficacy of Donor-Derived T Cells: Promising Potential and Pitfalls

Since the first clinical trials for EBV, CMV and AdV [47,48], the use of adoptive T cell therapy has rapidly grown, with high rates of response reported for VSTs. There has been a lack of large prospective multicenter clinical trials, and efficacy of virus-specific adoptive therapy has been difficult to quantify due to variations in manufacturing technology and patients treated. Indeed, endpoints of reported studies may differ (i.e., reduction in viral load, viral clearance, mortality), and differences in terms of efficacy between different strategies of manufacturing cannot be easily elucidated or reconciled. However, results have been consistent on the use of anti-CMV T cells as prophylaxis showing a beneficial effect on the occurrence of viral reactivation after HSCT [36,49], being significantly lower than in control groups. Clearance of CMV in refractory infections is expected in around 70–80% of patients [50,51,52]. Similarly, efficacy of anti-AdV and anti-EBV VSTs in terms of viral load clearance/reduction has been reported to be around 70% [39,53], and CTLs for EBV and AdV infection have been proved to be an effective prophylactic/pre-emptive therapy or treatment for PTLD and AdV reactivation post-HSCT. Especially in EBV-PTLD, CTLs are considered as second-line therapy after Rituximab [53]. Interestingly, despite the potential risk of inducing GvHD, its incidence was not increased when CTL recipients were compared with patients that did not received T cell infusions. Most authors have reported only minor-grade (I–II) GvHD, or minimal worsening of previous GVHD, associated with infusion of antigen-specific CTL, reflecting the high specificity of these cellular products and the efficiency of negative selection of alloreactive T cells. Although the cumulative incidence of acute and chronic GvHD did not differ from a control cohort [36], occasional severe (> grade II) acute GvHD has been reported in isolated cases [46]. Uses of selective CTLs to treat BK virus and JC virus reactivation have been reported as case reports, and no conclusions can be drawn on the efficacy of this approach [42]. The specificity of these products has also been demonstrated using an in vitro model of GvHD and anti-CMV T cells, which only demonstrated a grade I–II histopathological reaction on patient skin following patient–donor mixed lymphocyte reactions [54].

There are no accepted predictive markers for response; peripheral blood viral load is not a reliable parameter in the early stages, since EBV DNA can be undetectable despite ongoing lymphoproliferative disease within organs, and, conversely, CMV and AdV DNA can show transient surges early after T cell infusions, possibly due to the cytolysis of infected cells. 

Positive outcomes for refractory viral infection treated with virus-specific T cells from the HSCT donor can be hampered by several factors. One of the main variables influencing the curative potential of CTLs is appropriate timing for infusion. Different studies reported a significantly lower success rate when CTLs were administered in patients with organ damage or multiorgan involvement [34,46], although viral load clearance and positive outcome has been reported even in cases of severely ill patients (i.e., CMV encephalitis). Mortality due to viral infection is usually extremely high in patients that fail to respond to adoptive therapy [51], probably reflecting the advanced stage of disease at the time of cellular therapy. On the other hand, some authors suggest that in theory, infusion of CTLs may initially increase inflammation in involved tissues leading to additional organ damage and death [55], despite viral clearance. For these reasons, although an appropriate window of treatment cannot be defined, adoptive therapy should probably be used in the early phases of viral disease.

CTL effect on viral replication is mainly related to appropriate expansion of infused cells, which generally occurs within 2–4 weeks, but has been reported up to 16 weeks after infusion [51]. However, it can be difficult to predict the timing of adoptive T cell expansion due to several variables relating to the patient and transplant characteristics: initial viral load; ongoing immunosuppression; and invivo T cell depletion, conditioning, and persistence of serotherapy [39]. Theoretically, efficacy of CTLs could be hampered by use of concomitant immunosuppression, as is usual at this time point in the transplant setting. Most investigators prefer prednisone to be at a dose of <1 mg/kg at the time of CTL infusion, however, in some studies, patients receiving steroids and/or other immunosuppressive drugs showed similar response rates to patients without ongoing immunosuppression [53]. 

Persistence of cells is not evenly reported by authors, and possible differences could be related to manufacturing protocols, and immunosuppressive drugs used in the context of GvHD. Although infused CTLs have been detected more than eight years after the infusion [35], viral clearance is mainly related to early expansion of infused CTLs, more than the long-term persistence of adoptive cells in peripheral blood. Accordingly, the phenotypic composition of adoptive T cells can influence virus-specific T cell expansion. Terminally differentiated T cells show a lower persistence and efficacy when compared to less differentiated cells [56].

A minimum infusion dose is not easily determinable, since even a few cells, with large expansion potential, can lead to sustained antiviral immunity. It is also noteworthy that nonresponder patients who did not clear or significantly reduce the blood viral load can show appropriate cell expansion [57], again suggesting that outcomes after CTLs are not easily predictable. Interestingly, no significant differences in efficacy have been demonstrated with different CTL doses or administration schedules, even when compared to DLIs. Despite a narrow range of administered doses (1 × 10^4^/kg^−2^ × 10^6^/kg), even at the lowest of these doses, viral load clearance has been achieved, with a concomitant expansion of transferred T cells [35,53].

Treatment failures have been correlated to the lack of recognition of the target by infused CTLs [53]. T cells from the donor are sensitized in vitro to recognize specific HLA-restricted viral antigens. In the case of EBV-PTLD, when CTLs are sensitized using an exogenous strain, they may not be able to recognize the endogenous patient strain, which can present different antigen–HLA combinations to T cells. This may occur when mutation of the viral DNA leads to the lack of expression of certain epitopes or lack or recognition of specific HLA antigens by T cell receptor of donor T cells [53,58]. In selected scenarios, therefore, some patients could benefit from a personalized selection of CTLs derived from a third party that are able to recognize that specific HLA phenotype.

## 5. Third Party CTLs

Donor-derived VST infusions are not always feasible in clinical practice due to the lack of specific immunity of donor cells (e.g., high-risk patients: CMV-seropositive receiving HSCT from CMV-seronegative donor), or the availability of the donor (e.g., patients undergoing UCB transplantation), and the time required for manufacture of the cellular product might be too prolonged to deal with the rapid clinical evolution of viral infection. Moreover, treatment failure can occur due to specific HLA restriction of donor-derived CTLs that are unable to recognize viral epitopes generated by viral DNA mutation and presented by a different HLA complex. In this scenario, use of more specific HLA restriction of T cells could correctly recognize viral antigens and clear the virus [53]. Ideally, the use of selected T cells with higher grades of specificity for particular HLA types could add additional benefit in terms of viral clearance, minimizing the risk of treatment failure.

**Figure 1:**
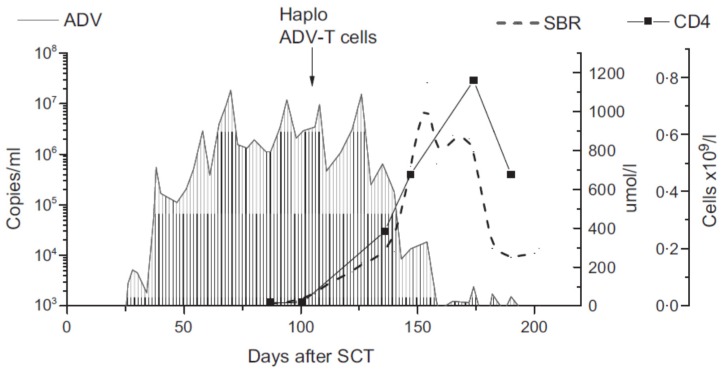
Third-party CTL efficiently cleared viral load in a transplanted child with refractory AdV infection, although significant liver GvHD occurred afterwards. Adapted from Qasim et al. [61].

The encouraging results following infusion of viral-specific T cells in solid organ-transplanted patients [59] suggested that third-party CTLs could be effectively used also in the setting of HSCT [53]. After a few initial reports [60,61] (Figure 1), demonstrating efficacy of third-party virus-specific T cells in HSCT patients, banks of third-party CTLs were developed in order to store specific antiviral T cell products that could be immediately available in the case of refractory infections. However, there are potential drawbacks to this approach: (1) partially matched cells might be recognized by the host immune system leading to rejection of CTLs; (2) the risk of alloreactive phenomena against recipient cells. Although few centers have implemented an “off-the-shelf” program of CTLs for HSCT recipients, and no randomized trials have been conducted yet, the published results suggest an overall response rate around 60–80% [62,63], both as prophylaxis and therapy, with a relatively low burden of severe complications (GvHD > grade II), similarly to donor-derived CTLs. This approach may be clinically beneficial in the early phases of post-transplant refractory infection, but lack of persistence of third party CTLs represents a concern, and there are still few data on long-term effects on viral clearance and specific immune reconstitution. Third-party CTLs have been detected up to 90 days after infusion, but persistence is expected to be shorter. Intriguingly, viral clearance is usually sustained, possibly related to specific antiviral immunity recovery following CTL infusion [63]. As mentioned above, third-party CTLs could also represent a promising alternative in patients who fail to respond to donor-derived CTLs and present viral genome mutations that lead to specific antigen–HLA combinations that are unrecognized by donor T cells. A more complete understanding of the mechanism of viral recognition by CTLs could lead to a personalized approach to adoptive cell therapy by matching the recipient HLA–epitope combination with the more appropriate HLA-restricted CTLs.

## 6. Conclusions

Viral infections after HSCT can be challenging for clinicians, and are a major cause of morbidity and mortality, especially in high-risk patients. Infusion of adoptive VSTs can tackle viral replication and restore antiviral immunity in patients not responding to antiviral drugs. The expected response rates are between 50–90%, with different manufacturing techniques, and even in the HLA partially matched setting, antiviral efficacy is reported around 50–70%. Toxicity is usually temporary and easily controlled, occurring only in a small proportion of patients. Further challenges might be addressed by broadening applicability to all transplanted patients (i.e., patients with active GvHD) and reducing the time to access cellular products using VST cell banks. Moreover, transplant-related mortality also arises from respiratory virus infections, JC and BK virus, and other viral infections that lack an effective antiviral treatment. Therefore, cellular products that can also target these viruses are required. Future challenges of adoptive therapies will include broadening applicability to different categories of patients, and the better definition of patient-oriented strategies.

## Figures and Tables

**Table 1:** Reported incidence of AdV, CMV and EBV post-transplant reactivation in peripheral blood, and disease-specific pharmacological treatment and rate of treatment response in children and adults undergoing haematopoietic stem cell transplantation.

Patients	Viremia	Viral Disease	Treatment	Response Rate
**AdV**				
Children	15–30%	6–11%	Cidofovir, brincidofovir	60–80%
Adults	6–15%	2%		
**CMV**				
Children	15–20%	4%	Gancyclovir, foscarnet, valgancyclovir	70–80%
Adults	39%	13%		
**EBV**				
Children	11%	1–7%	Rituximab	60–70%
Adults	22%	1–3%		

**Table 2:** Largest published clinical trials for treatment and prevention of viral reactivation after HSCT using donor-derived single-virus adaptive therapy, using either ex vivo expansion or direct selection.

Patients (*n*)	Population	Viral Infection	VST Stimulated or Isolated by	Citation
Ex-Vivo Expanded				
113	Adults/Children	EBV	LBC	Heslop, 2010 [35]
50	Adults/Children	CMV	Mo-DC pp65-restricted	Blyth, 2013 [36]
8	Children	ADV	Multi-peptides AdV5	Ip, 2018 [37]
Direct Selection				
10	Adults/Children	EBV	IFN-γ capture	Icheva, 2013 [38]
18	Unknown	CMV	IFN-γ capture	Peggs, 2011 [34]
30	Adults/Children	ADV	IFN-γ capture	Feucht, 2015 [39]

Abbreviations: LBC: lymphoblastoid cells; IFN: interferon; Mo-DC: monocyte-derived dendritic cells.

**Table 3:** Largest published clinical trials for treatment and prevention of viral reactivation after HSCT using donor-derived multiple-virus adaptive therapy.

Patients (*n*)	Population	Viral Specificity	VST Stimulated by	Citation
13	Children	EBV/ADV	LBCs transduced with ADV vector	Leen, 2009 [45]
15	Adults/Children	ADV/CMV/EBV	LBCs transduced with ADV vector encoding CMVpp65	Leen, 2006 [32]
10	Adults	ADV/CMV/EBV/VZV	Mo-DC transduced with ADV vector encoding CMVpp65, EBNA1, VZV vaccine	Ma, 2015 [46]
11	Adults/Children	ADV/CMV/EBV/BKV/HHV6	Pepmixes of immunodominant antigens	Papadopulou, 2014 [43]

Abbreviations: LBC: lymphoblastoid cells; Mo-DC: monocyte-derived dendritic cells; VZV: Varicella–Zoster virus; EBNA1: Epstein–Barr nuclear antigen 1.
